# Occult mediastinal lymph node metastasis in FDG‐PET/CT node‐negative lung adenocarcinoma patients: Risk factors and histopathological study

**DOI:** 10.1111/1759-7714.13093

**Published:** 2019-05-24

**Authors:** Huang Miao, Li Shaolei, Li Nan, Lai Yumei, Zhang Shanyuan, Lu Fangliang, Yang Yue

**Affiliations:** ^1^ Department of Thoracic Surgery II, Key Laboratory of Carcinogenesis and Translational Research (Ministry of Education) Peking University Cancer Hospital and Institute Beijing China; ^2^ Department of Nuclear Medicine, Key Laboratory of Carcinogenesis and Translational Research (Ministry of Education) Peking University Cancer Hospital and Institute Beijing China; ^3^ Department of Pathology, Key Laboratory of Carcinogenesis and Translational Research (Ministry of Education) Peking University Cancer Hospital and Institute Beijing China

**Keywords:** Adenocarcinoma, lung cancer, mediastinal lymph node, metastasis, positron emission tomography

## Abstract

**Background:**

The aim of this study was to investigate predictive factors of occult mediastinal lymph node metastasis (MLNM) in preoperative ^18^F‐fluorodeoxy‐glucose PET/CT node‐negative lung adenocarcinoma patients.

**Methods:**

We reviewed the clinical data and PET/CT parameters of 360 consecutive pulmonary adenocarcinoma patients who were scheduled to undergo anatomical pulmonary resection and systemic mediastinal node dissection. The nodal metastasis was pathologically defined and all resected tumors were classified according to the 2011 IASLC/ATS/ERS classification. Univariate and multivariate analysis were conducted to evaluate the associations between clinicopathological variables and MLNM.

**Results:**

Of all 360 patients, 54 (15.0%) had pathological N2 diseases. The serum CEA level, nodule type, hilar nodal SUVmax, tumor SUVmax, size, location and histologic subtype were associated with MLNM significantly on univariate analysis. On multivariate analysis, CEA ≥ 5.0 ng/mL (*P* < 0.001), solid nodule (*P* = 0.012), tumor SUVmax ≥ 3.7 (*P* < 0.027), hilar nodal SUVmax ≥ 2.0 (*P* < 0.001) and centrally located tumor (*P* = 0.035) were independent risk factors for MLNM. The area under the ROC curve (AUC) for tumor SUVmax and hilar nodal SUVmax in predicting MLNM was 0.764 and 0.730, respectively, and the combined use of five factors yielded a higher AUC of 0.885.

**Conclusion:**

Increased primary tumor and hilar lymph node SUVmax, solid nodule, centrally located tumor and increased CEA level predicted the increased risk of mediastinal lymph node metastasis. Combined use of these factors improved the diagnostic capacity for predicting N2 disease preoperatively. Invasive mediastinal staging should be considered for patients with these risk factors, even those with a negative mediastinum on PET/CT.

## Introduction

Lung cancer is the leading cause of cancer‐related death worldwide, and non‐small cell lung cancer (NSCLC) accounts for 80% of cases.^1^ Adenocarcinoma is the most common subtype of surgically resectable NSCLC. In patients with pathologically documented mediastinal lymph node metastasis (MLNM), the role of surgery remains controversial.^2^ The presence of N2 disease indicates a poor prognosis, and it is recommended that patients undergo neoadjuvant therapy before anatomical resection.^3^ Thus toaccurately predict mediastinal lymph node status is critical for assessing prognosis and selecting the optimal therapy.

^18^F‐fluorodeoxy‐glucose (FDG) positron emission tomography is a functional imaging modality that is based on the increased glucose metabolism of malignant cells.^4^ The maximum standardized uptake value (SUVmax) is the most representative marker of FDG uptake and is widely used. Over the past decade, ^18^F‐FDG PET combined with CT (PET/CT) has become the standard method for non‐invasive lymph node staging. However, the low sensitivity and high false‐negative rate for predicting MLNM of PET/CT has limited its use for staging.^5^ A recent meta‐analysis showed that the pooled sensitivity of PET/CT in identifying MLNM was 68%, which has resulted in many nodal micrometastasis going undetected, and the incidence of occult MLNM in patients with negative nodal uptake on PET/CT was 7–18%.^6^ Thus part of the major attention has been shifted from FDG uptake of mediastinum to primary tumor and hilar lymph nodes, especially for PET N2 node‐negative cases, as a few publications have reported that tumor SUVmax was correlated with mediastinal lymph node metastasis.^7^, ^8^ However, few studies have reported the diagnostic value of tumor and hilar lymph nodal FDG uptake in predicting occult N2 involvement.

Most studies have focused on identifying high‐risk factors for mediastinal nodal involvement in clinical N2‐negative patients, which include gender, smoking status, tumor location, tumor size, histologic type, CEA level and tumor differentiation.^9^, ^10^ As more and more attention was given to the histologic features of lung cancers to stratify patients more accurately, the International Association for the Study of Lung Cancer, American Thoracic Society and European Respiratory Society (IASLC/ATS/ERS) developed the new classification of lung adenocarcinoma.^11^ Various studies have proven this new classification as an independent predictor of clinical outcomes.^12^, ^13^ The predominant histologic subtypes of lung adenocarcinoma are associated with occult lymph node metastasis.^14^ However, the relationship between tumor FDG uptake with histologic subtypes and their combined predictive value for N2 disease has not been fully interpreted.

In this study, we investigated the predictive factors including PET/CT parameters for MLNM in a consecutive surgical resected lung adenocarcinoma cohort. The diagnostic value of tumor SUVmax in predicting occult MLNM was evaluated and its relationship with corresponding histologic subtype analyzed. Moreover, combined use of these factors was conducted to improve the diagnostic capability in predicting occult MLNM.

## MethodsPatients selection and staging

We retrospectively reviewed patients with diagnosed lung adenocarcinoma who had undergone surgery on curative purpose from May 2012 to December 2015 at the department of Thoracic Surgery II, Peking University Cancer Hospital. Three hundred and sixty consecutive patients with histologically proven lung adenocarcinoma underwent staging with integrated PET/CT prior to lung resection were enrolled in this study. Patients who received preoperative chemotherapy or radiotherapy and those with evidence of FDG‐avid mediastinal nodal disease were excluded. Other preoperative tests were routinely performed as follows: enhanced chest CT, enhanced brain magnetic resonance imaging and laboratory tests including serum carcinoembryonic antigen (CEA) level. Preoperative serum CEA levels were assessed using enzyme‐linked immunosorbent assay kits, with an upper limit of normal of 5.0 ng/mL. This retrospective study was approved by the Ethics Committee of Beijing Cancer Hospital. Written informed consent was obtained from all patients which contained a statement to approve their disease information could be used for research purposes. Preoperative clinical staging and postoperative pathological staging were based on the TNM Classification of Malignant Tumors, 8th Edition.

### Integrated PET/CT imaging

Preoperative PET/CT was performed using a Gemini TF PET/CT system (Philips). After fasting for at least six hours and resting for 60 minutes, an intravenous injection of 3.7 MBq of 18FDG/kg bodyweight was administered. PET/CT data was obtained from patients in the supine position. Emission images were acquired after CT scanning, and an emission scan was performed in eight to10 bed positions with one minute per step. The FDG uptake of tumor was visually compared with that of the surrounding tissue in areas devoid of prominent artifacts and overlapping increased FDG uptake organs. A team of experienced radiologists reviewed the integrated PET/CT images independently. The SUVmax of the primary tumor and SUVmax of hilar lymph nodes were recorded, respectively. A tumor was defined central if its center was located in the inner one third of the lung parenchyma on transverse CT imaging and peripherally located tumors were identified as those centered in the outer two thirds. Definitions of solid or subsolid nodules used in the classification of lung adenocarcinoma were based on the Fleishner Society glossary of terms.^15^ All integrated PET/CT imaging was performed within fourweeks before surgery.

### Surgical resection

All the surgical procedures including pulmonary resection and lymph node dissection were conducted by experienced thoracic surgeons at the Department of Thoracic Surgery II of Peking University Cancer Hospital. All patients received video‐assisted thoracoscopic surgeries (VATS) and the procedures performed included anatomical lobectomy and pneumonectomy. All patients received systemic mediastinal lymphadenectomy in accordance with the International Association for the Study of Lung Cancer (IASLC) proposals,^16^ which suggested dissection of lymph nodes from stations 2R, 4R, 7, 10R and 11R for right‐sided tumors, and stations five, six, seven, 10L and 11L for left‐sided tumors.

### Pathological examination

All resected specimens were sent for routine pathologic analysis and examined histologically by experienced pulmonary pathologists. Histological classification of the tumor was based on the new IASLC/ERS/ATS classification of pulmonary adenocarcinoma.^11^ Each histological component—lepidic, papillary, acinar, mucinous, micropapillary and solid pattern—was recorded if ≥5% of the histological pattern was presented and tumors were classified according to predominant patterns. The dissected lymph nodes were examined histologically following hematoxylin and eosin staining.

### Statistical analysis

All Statistical analysis was performed using SPSS software package (version 22.0; SPSS, Chicago, IL, USA). Pearson's chi‐square test or Fisher's exact test were used to test univariate associations between lymph node metastasis and clinicopathological factors. Variables that were significant on univariate analysis were also included in the multivariable logistic regression analysis to evaluate independent risk factors for predicting MLNM. The receiver‐operating characteristic (ROC) curve was performed to obtain the SUVmax cutoff value and the area under the ROC curve (AUC) was used to assess the predictive value of established criteria. Subsequently, diagnostic parameters such as sensitivity, specificity, positive predictive value (PPV), negative predictive value (NPV) and accuracy were determined by diagnostic test fourfold table. Tumor SUVmax among histologic subtypes were compared using the nonparametric Kruskal‐Wallis test, and followed by the Bonferroni‐Dunn *post hoc* test for the difference between two subtypes. *P*‐values were considered statistically significant at *P* < 0.05.

## Results

### Patient characteristics and incidence of lymph node metastasis

The characteristics of patients enrolled in this study are shown in Table 1. Of the 360 patients who were evaluated retrospectively, most were female (*n* = 216, 60%) and had never smoked (*n* = 251, 69.7%), with a mean age of 61 ± 9 years. The mean tumor size was 2.6 ± 1.2 cm and most of the evaluated nodules were solid (*n* = 220, 61.1%). Based on the IALSC/ERS/ATS classification, 2 (0.6%) of the 360 tumors were reclassified into adenocarcinoma *in situ* (AIS), 4 (1.1%) were microinvasive adenocarcinoma (MIA), 47 (13.1%) were lepidic predominant adenocarcinoma (LPA), 52 (14.1%) were papillary predominant adenocarcinoma (PPA), 208 (57.8%) were acinous predominant adenocarcinoma (APA), 10 (2.8%) were invasive mucinous adenocarcinoma (IMA), 6 (1.7%) were micropapillary predominant adenocarcinoma (MPA) and 31 (8.6%) were solid predominant adenocarcinoma (SPA). Two hundred and fifty five (70.8%) of the tumors consisted of mixed subtypes with at least two growth patterns. Of all the 360 patients, 54 (15.0%) had MLNM. The mean number of mediastinal lymph nodes dissected was 10.15 ± 6.04. A total of 1345 N2 stations (3654 lymph nodes) were sampled or dissected and 100 (7.4%) stations were pathologically proved positive.

**Table 1:** Characteristics of patients and tumors (*n* = 360)

Characteristics	Distribution (%)
Age (years)	
Median	60
Mean ± SD	61 ± 9
Range	32–86
Sex	
Female	216 (60.0)
Male	144 (40.0)
Smoking status	
Never	251 (69.7)
Current or former	109 (30.3)
CEA (ng/mL)	
<5.0	282 (78.3)
≧5.0	78 (21.7)
Nodule type	
Subsolid nodule	140 (38.9)
Solid nodule	220 (61.1)
Tumor size on CT (cm)	
Median	2.3
Mean ± SD	2.6 ± 1.2
Range	0.3–8.4
Tumor SUVmax	
Median	4.29
Mean ± SD	5.18 ± 3.70
Range	0.00–22.70
Tumor location	
Central	93 (25.8)
Peripheral	267 (74.2)
Histologic subtype	
Adenocarcinoma in situ	2 (0.6)
Microinvasive adenocarcinoma	4 (1.1)
Lepidic predominant adenocarcinoma	47 (13.1)
Papillary predominant adenocarcinoma	52 (14.4)
Acinous predominant adenocarcinoma	208 (57.8)
Invasive mucinous adenocarcinoma	10 (2.8)
Micropapillary predominant adenocarcinoma	6 (1.7)
Solid predominant adenocarcinoma	31 (8.6)
Pathological N stage	
N0	264 (73.3)
N1	42 (11.7)
N2	54 (15.0)
TNM stage	
0	2 (0.6)
IA	154 (42.8)
IB	93 (25.8)
IIA	7 (1.9)
IIB	41 (11.4)
IIIA	60 (16.7)
IIIB	3 (0.8)

SD, standard deviation; SUVmax, maximum standardized uptake value.

### Clinicopathological factors associated with MLNM

**Figure 1:**
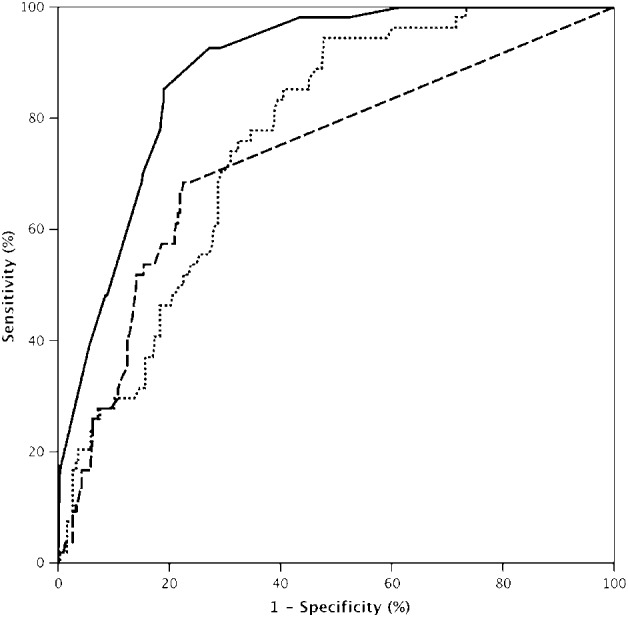
Comparative ROC curves of tumor SUVmax, hilar LN SUVmax and combined five factors (tumor SUVmax, hilar LN SUVmax, nodule classification, CEA level, central or peripheral located tumor) 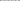 for predicting mediastinal lymph node metastasis. The cutoff value was 3.7 for tumor SUVmax, the sensitivity, specificity, positive predictive value (PPV), negative predictive value (NPV), accuracy were 94.4%, 52.0%, 25.8%, 98.1%, 58.3%, respectively, and the AUC was 0.764 (95%CI: 0.706–0.821). The cutoff value was 2.0 for hilar LN 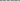 SUVmax, the sensitivity, specificity, PPV, NPV, accuracy were 68.5%, 77.4%, 34.9%, 93.3%, 76.1%, respectively, and the AUC was 0.730 (95%CI: 0.652–0.807). The AUC of combined predictive value of the five factors 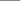 reached 0.885 (95%CI: 0.847–0.924).

The ROC curves are shown in Figure 1. The optimal cutoff values of tumor SUVmax and hilar lymph node (LN) SUVmax to predict MLNM, based on the Youden's index, were 3.7 and 2.0, respectively. We dichotomize the patients according to these thresholds. The results of the univariate analysis for clinicopathological factors associated with MLNM are presented in Table 2. The general characteristics including age, gender, smoking status and concurrent disease between patients with pathologic N0‐1 and patients with pathologic N2 were not significantly different. Factors significantly associated with MLNM are: CEA ≥ 5.0 ng/mL (*P* < 0.001), tumor size (*P* = 0.033), solid nodule (*P* < 0.001), tumor SUVmax ≥ 3.7 (*P* < 0.001), hilar LN SUVmax ≥ 2.0 (*P* < 0.001), central located tumor (*P* = 0.005). Examination of histological factors revealed that specific predominant subtypes were associated with N2 disease (*P* < 0.001). Additionally, the absence of lepidic pattern (*P* < 0.001), the presence of micropapillary pattern (*P* = 0.024) and the presence of solid pattern (*P* = 0.022) were significantly associated with an increased risk of N2 disease.

**Table 2:** Univariate analysis for clinicopathological factors associated with mediastinal lymph node metastasis

Variables	Pathological N0‐1	Pathological N2	*P‐*value
	*n* (%)	*n* (%)	
Age (years)†	60.5 ± 9.6	60.7 ± 8.3	0.857
Sex			
Female	183 (84.7)	33 (15.3))	
Male	123 (85.4)	21 (14.6)	0.857
Smoking status			
Never	216 (86.1)	35 (13.9)	
Current or former	90 (82.6)	19 (17.4)	0.395
CEA (ng/mL)			
<5.0	259 (91.8)	23 (8.2)	
≧5.0	47 (60.3)	31 (39.7)	<0.001
Tumor size on CT (cm)†	2.5 ± 1.2	2.9 ± 1.3	0.033
Nodule type			
Subsolid nodule	137 (97.9)	3 (2.1)	
Solid nodule	169 (76.8)	51 (23.2)	<0.001
Tumor SUVmax			
<3.7	159 (98.1)	3 (1.9)	
≥3.7	147 (74.2)	51 (25.8)	<0.001
Hilar LN SUVmax			
<2.0	237 (93.3)	17 (6.7)	
≥2.0	69 (65.1)	37 (34.9)	<0.001
Tumor location			
Central	71 (76.3)	22 (23.7)	
Peripheral	235 (88.0)	32 (12.0)	0.005
Predominant subtype			
Lepidic	53 (100.0)	0 (0.0)	
Papillary	49 (94.2)	3 (5.8)	
Acinar	174 (83.7)	34 (16.3)	
Mucinous	7 (70.0)	3 (30.0)	
Micropapillary	4 (66.7)	2 (33.3)	
Solid	19 (61.3)	12 (38.7)	<0.001
Lepidic pattern			
Absent	196 (79.7)	50 (20.3)	
Present	110 (96.5)	4 (3.5)	<0.001
Papillary pattern			
Absent	191 (83.0)	39 (17.0)	
Present	115 (88.5)	15 (11.5)	0.167
Acinar pattern			
Absent	66 (83.5)	13 (16.5)	
Present	240 (85.4)	41 (14.6)	0.682
Invasive mucinous pattern			
Absent	292 (85.6)	49 (14.4)	
Present	14 (73.7)	5 (26.3)	0.181
Micropapillary pattern			
Absent	280 (86.4)	44 (13.6)	
Present	26 (72.2)	10 (27.8)	0.024
Solid pattern			
Absent	251 (87.2)	37 (12.8)	
Present	55 (76.4)	17 (23.6)	0.022

LN, lymph node; SUVmax, maximum standardized uptake value.

† Values are mean ± standard deviation.

Multivariate logistic regression analysis, as shown in Table 3, revealed that CEA ≥ 5.0 ng/mL (*P* < 0.001), solid nodule (*P* = 0.012), tumor SUVmax ≥ 3.7 (*P* = 0.027), hilar LN SUVmax ≥ 2.0 (*P* < 0.001) and centrally located tumors (*P* = 0.035) were independent risk factors associated with MLNM. The odds ratios of the five factors are presented in Table 3.

**Table 3:** Multivariate regression analysis for factors associated with mediastinal lymph node metastasis

Variables	Odds ratio	Confidence interval	*P‐*value
CEA ≧ 5 ng/mL	3.70	1.80–7.61	<0.001
Tumor size	0.92	0.69–1.22	0.559
Solid nodule	6.58	1.52–28.37	0.012
Tumor SUVmax ≧ 3.7	4.51	1.19–17.10	0.027
Hilar LN SUVmax ≧ 2.0	6.66	3.07–14.43	<0.001
Central location	2.32	1.06–5.06	0.035
Lepidic pattern present	1.02	0.27–3.80	0.978
Micropapillary pattern present	1.85	0.69–4.93	0.222
Solid pattern present	0.75	0.34–1.66	0.476

LN, lymph node; SUVmax, maximum standardized uptake value.

### Association of tumor SUVmax with various histologic subtypes

**Figure 2:**
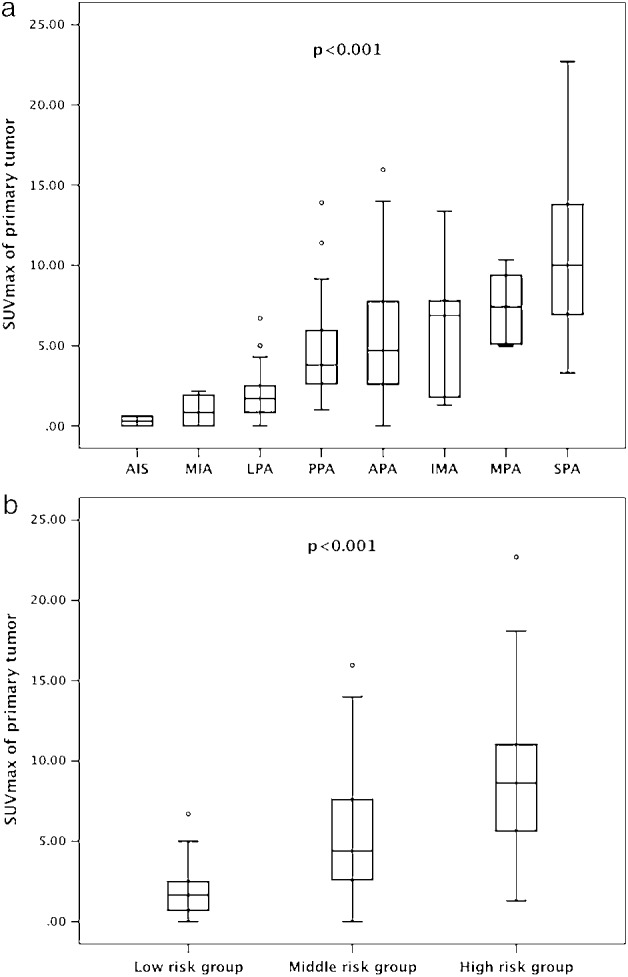
(**a**) Tumor SUVmax (mean ± SD) in each histologic subtypes were as follows: AIS, 0.30 ± 0.42; MIA, 0.97 ± 1.13; LPA, 1.83 ± 1.46; PPA, 4.60 ± 2.72; APA, 5.37 ± 3.20; IMA, 5.88 ± 3.95; MPA, 7.44 ± 2.39 and SPA, 10.26 ± 4.63. The *P*‐value determined by the Kruskal‐Wallis test was <0.001. (**b**) Tumor SUVmax (mean ± SD) in different risk groups were as follows: low risk group: AIS+MIA+LPA, 1.71±1.45; PPA+APA, 5.21 ± 3.12; IMA+MPA+SPA, 8.97 ± 4.60. The *P*‐value determined by the Kruskal‐Wallis test was <0.001. AIS, adenocarcinoma *in situ*; APA, acinar predominant invasive adenocarcinoma; IMA, invasive mucinous adenocarcinoma; LPA, lepidic predominant invasive adenocarcinoma; MIA, minimally invasive adenocarcinoma; MPA, micropapillary predominant invasive adenocarcinoma; PPA, papillary predominant invasive adenocarcinoma; SPA, solid predominant invasive adenocarcinoma.

Histologic subtypes of adenocarcinoma based on the new IALSC/ERS/ATS classification were proved significant factors for predicting MLNM on univariate analysis as above. However, none were independently associated with MLNM on multivariate analysis. As shown in Figure 2a, tumor SUVmax among histologic subtypes differed significantly using the Kruskal‐Wallis test (*P* < 0.001). Significant differences were found using the Bonferroni‐Dunn test in pairwise comparisons: LPA and PPA (*P* < 0.001), LPA and APA (*P* < 0.001), LPA and IMA (*P* = 0.006), PPA and SPA (*P* < 0.001), APA and SPA (*P* < 0.001). When patients were classified into three risk groups according to Yoshizawa *et al.* 12: AIS+MIA+LPA (low risk), PPA+APA (middle risk), IMA+MPA+SPA (high risk), the differences of tumor SUVmax were more remarkable (*P* < 0.001, Fig 2b).

### Diagnostic value of using combinations of risk factors to predict MLNM

The predictive values for MLNM of tumor SUVmax and hilar LN SUVmax quantified by AUC of ROC analysis were 0.764 and 0.730, respectively, as shown in Figure 1. The diagnostic capacity of each one parameter alone is not sufficiently powerful and confident enough for predicting MLNM. However, the integration of the five independent risk factors (tumor SUVmax, hilar LN SUVmax, nodule classification, CEA level, central or peripheral location) together in a multivariate logistic regression equation could obtain a new probability value for each case, and we got a combined ROC curve subsequently for diagnosing N2 disease (Fig 1). The AUC was profoundly increased to 0.885, which exhibited a higher and considerable diagnostic value.

## Discussion

In this study, we investigated various clinicopathological factors that may be associated with MLNM in a consecutive lung adenocarcinoma cohort. Our study demonstrated that the primary tumor SUVmax was an independent risk factor for predicting MLNM in lung adenocarcinoma. A previous study reported that the SUVmax of primary NSCLC correlated with lymph node metastasis and could predict survival.^17^ However, no detailed mechanism has as yet been found to explain this phenomenon. A study by Li *et al.* 18 reported that the probability of LNM increased with each unit increase in SUVmax of primary NSCLC and Sachs *et al.* 19 confirmed this hypothesis. Our study obtained similar results. In addition, we evaluated the diagnostic value of primary tumor as well as hilar LN SUVmax for predicting MLNM and they were also shown to be important predictive factors. We also attempted to explore the underlying mechanism of this phenomenon by analyzing the histologic features.

The 2011 IASLC/ATS/ERS classification system for lung adenocarcinoma, based on classification by predominant patterns, has been shown as a good tool to predict clinical outcomes in many recent studies. Several publications showed that micropapillary and solid patterns were associated with aggressive biological behaviors, such as lymphovascular invasion, pleural invasion, lymph node metastasis, advanced stage and poor prognosis.^20^, ^21^ Sica *et al.* 22 compared the histologic subtypes between primary tumor and metastatic sites and found that when the primary tumor was composed predominantly of either micropapillary or solid types, the tumor at the metastatic site presented the same subtype in 86% to 100% of the cases. The presence of alepidic pattern was shown to correlate with a more favorable outcome.^23^

Our findings, although obtained from retrospective data analysis, were concordant with previous studies. The results of univariate analysis revealed that the absence of lepidic pattern, the presence of micropapillary pattern and the presence of solid pattern were significantly associated with an increased risk of MLNM. However, on multivariate analysis, when the PET/CT parameters were also included, none of the growth patterns of adenocarcinoma were significantly associated with MLNM. This confounding phenomenon urged us to explore the relationship of tumor SUVmax between histologic subtypes. As expected, significant differences were found among different subtypes and different risk groups. Thus, the tumor SUVmax correlated with histologic subtypes closely, and histologic subtypes correlated with nodal status closely which has been proved by this study and those previously published.^13^, ^14^ The close associations with histologic subtypes may underlie the mechanism of tumor SUVmax for predicting MLNM in lung adenocarcinoma.

Another important risk factor revealed in our study was the CT appearance of primary tumor. According to the Fleishner Society,^15^ pulmonary nodules were classified into solid and subsolid nodules. A solid nodule, which was defined by a focal area of high attenuation that completely obscures the lung parenchyma within, was also proved an independent risk factor for MLNM. Other than tumor size, the radiological parameters such as ground‐glass opacity (GGO) ratio, tumor disappearance rate and consolidation diameter in thin‐section CT, are attracting more and more attention. Several recent studies report that the much more solid components the nodule presented, the worse the prognosis is.^24^, ^25^ On the contrary, a subsolid nodule was shown to correlate with low metastatic risk for containing ground glass components and pure GGO was rarely associated with lymph node metastasis. In fact, the pure GGO manifested most frequently as adenocarcinoma with lepidic predominant growth pattern microscopically.^26^ This was in agreement with our findings. It would be interesting to further investigate these radiological parameters quantitatively and their integration with histologic features could predict clinical outcomes more accurately.

Generally speaking, centrally located tumors tend to be associated with higher stages and a poor prognosis. Ketchedjian *et al.,* 27 reported that central tumors were more advanced and demonstrated a significantly (*P* < 0.001) poorer survival than peripheral tumors, because central tumors may harbor a higher incidence of occult nodal involvement. Many publications reported that serum CEA level was associated with the post‐operative pathological stage and prognosis. Inoue *et al.* 28 and Bao *et al.* 29 reported that increased CEA level was associated with a much higher rate of lymph node metastasis and a worse prognosis in small sized NSCLC. Centrally located tumors and high CEA levels were also proved independent risk factors for N2 disease in our study, although in lung adenocarcinoma patients, and still concordant with previous studies. Thus, care should be taken against mediastinal lymph node involvement in centrally located tumors or patients with high CEA levels.

The major strength of our study was that we established reliable clinical criteria for the prediction of MLNM according to the results of multivariate analysis. Taking into account five factors (tumor SUVmax, nodal SUVmax, nodule classification, CEA level, central or peripheral located tumor), a predictive value was achieved with a higher AUC of 0.885 for diagnosing N2 disease. Moreover, they were amenable to clinical use due to their non‐invasive nature. The more risk factors one patient has, the higher risk that he or she would have mediastinal involvement. For example when all the five risk factors are present, even with a negative mediastinum on PET, there is a 90% probability that he or she would have N2 disease, and invasive pathologic mediastinal staging method such as endobronchial ultrasonography (EBUS) or mediastinoscopy is strongly recommended. Neoadjuvant therapy should also be taken into consideration before surgery.

However, our study has several limitations. The first limitation is its retrospective, single‐institution nature, and the selection bias which could not be avoided. Moreover, the follow‐up time of our cohort is relatively short and the relationships of these factors with survival could not as yet be identified. Whether these predictive factors of MLNM are also predictive for further outcomes needs more observation time and a follow‐up report is expected.

## Conclusions

The primary tumor SUVmax, hilar lymph node SUVmax, solid nodule, centrally located tumor and increased CEA level were found independent risk factors for mediastinal lymph node metastasis in adenocarcinoma. Combined use of these factors improved the diagnostic capacity for predicting N2 disease preoperatively and invasive mediastinal staging should be strongly recommended in these patients, even those with a negative mediastinum on PET. The close association with histologic subtypes may underlie the mechanism of tumor SUVmax for predicting MLNM in lung adenocarcinoma. These previously unpublished observations may deepen our understanding of PET/CT parameters and have potential implications for therapeutic management of lung adenocarcinoma patients.
